# Decreased sphingomyelin (t34:1) is a candidate predictor for lung squamous cell carcinoma recurrence after radical surgery: a case-control study

**DOI:** 10.1186/s12885-021-08948-5

**Published:** 2021-11-17

**Authors:** Yusuke Takanashi, Kazuhito Funai, Fumihiro Eto, Kiyomichi Mizuno, Akikazu Kawase, Hong Tao, Takuya Kitamoto, Yutaka Takahashi, Haruhiko Sugimura, Mitsutoshi Setou, Tomoaki Kahyo, Norihiko Shiiya

**Affiliations:** 1 Department of Cellular and Molecular Anatomy, Hamamatsu University School of Medicine, 1-20-1 Handayama, Higashi Ward, Hamamatsu, Shizuoka, 431-3192 Japan; 2 First Department of Surgery, Hamamatsu University School of Medicine, 1-20-1 Handayama, Higashi Ward, Hamamatsu, Shizuoka, 431-3192 Japan; 3 Department of Tumor Pathology, Hamamatsu University School of Medicine, 1-20-1 Handayama, Higashi Ward, Hamamatsu, Shizuoka, 431-3192 Japan; 4 Advanced Research Facilities & Services, Hamamatsu University School of Medicine, 1-20-1 Handayama, Higashi Ward, Hamamatsu, Shizuoka, 431-3192 Japan; 5 Preppers Co. Ltd., 1-23-17 Kitashinagawa, Shinagawa Ward, Tokyo, 140-0001 Japan; 6 International Mass Imaging Center, Hamamatsu University School of Medicine, 1-20-1 Handayama, Higashi Ward, Hamamatsu, Shizuoka, 431-3192 Japan; 7 Department of Systems Molecular Anatomy, Institute for Medical Photonics Research, Hamamatsu University School of Medicine, 1-20-1 Handayama, Higashi Ward, Hamamatsu, Shizuoka, 431-3192 Japan

**Keywords:** Lung squamous cell carcinoma, Prognostic factor, Recurrence prediction, Lipid, Mass spectrometry

## Abstract

**Background:**

To reduce disease recurrence after radical surgery for lung squamous cell carcinomas (SQCCs), accurate prediction of recurrent high-risk patients is required for efficient patient selection for adjuvant chemotherapy. Because treatment modalities for recurrent lung SQCCs are scarce compared to lung adenocarcinomas (ADCs), accurately selecting lung SQCC patients for adjuvant chemotherapy after radical surgery is highly important. Predicting lung cancer recurrence with high objectivity is difficult with conventional histopathological prognostic factors; therefore, identification of a novel predictor is expected to be highly beneficial. Lipid metabolism alterations in cancers are known to contribute to cancer progression. Previously, we found that increased sphingomyelin (SM)(d35:1) in lung ADCs is a candidate for an objective recurrence predictor. However, no lipid predictors for lung SQCC recurrence have been identified to date. This study aims to identify candidate lipid predictors for lung SQCC recurrence after radical surgery.

**Methods:**

Recurrent (*n* = 5) and non-recurrent (*n* = 6) cases of lung SQCC patients who underwent radical surgery were assigned to recurrent and non-recurrent groups, respectively. Extracted lipids from frozen tissue samples of primary lung SQCC were analyzed by liquid chromatography-tandem mass spectrometry. Candidate lipid predictors were screened by comparing the relative expression levels between the recurrent and non-recurrent groups. To compare lipidomic characteristics associated with recurrent SQCCs and ADCs, a meta-analysis combining SQCC (*n* = 11) and ADC (*n* = 20) cohorts was conducted.

**Results:**

Among 1745 screened lipid species, five species were decreased (≤ 0.5 fold change; *P* < 0.05) and one was increased (≥ 2 fold change; *P* < 0.05) in the recurrent group. Among the six candidates, the top three final candidates (selected by AUC assessment) were all decreased SM(t34:1) species, showing strong performance in recurrence prediction that is equivalent to that of histopathological prognostic factors. Meta-analysis indicated that decreases in a limited number of SM species were observed in the SQCC cohort as a lipidomic characteristic associated with recurrence, in contrast, significant increases in a broad range of lipids (including SM species) were observed in the ADC cohort.

**Conclusion:**

We identified decreased SM(t34:1) as a novel candidate predictor for lung SQCC recurrence. Lung SQCCs and ADCs have opposite lipidomic characteristics concerning for recurrence risk.

**Trial registration:**

This retrospective study was registered at the UMIN Clinical Trial Registry (UMIN000039202) on January 21, 2020.

**Supplementary Information:**

The online version contains supplementary material available at 10.1186/s12885-021-08948-5.

## Background

Lung cancer remains one of the leading causes of cancer-related mortalities worldwide [1], and non-small cell lung cancer (NSCLC) accounts for 85% of all lung cancer cases [2]. The standard treatment for resectable NSCLC of stage I-III is radical resection [3]. Among them, induction chemoradiotherapy or adjuvant chemotherapy can be treatment options for advanced-stage NSCLC [4–7]. Adjuvant chemotherapy is recommended to reduce the risk of lung cancer recurrence after radical surgery [5–7]. Nonetheless, prognosis after radical surgery could be improved since up to 23.9% of patients who received radical surgery experience local or distant disease recurrence [8].

Identifying patients at high risk for recurrence who are likely to benefit from adjuvant chemotherapy will improve prognosis after radical surgery. Conversely, identifying patients at low risk for recurrence in whom the adverse events associated with adjuvant chemotherapy outweigh its benefit will enable cutting off unnecessary adjuvant chemotherapy [6, 7]. Those at high risk of recurrence should undergo adjuvant chemotherapy, while the low risk-patients should be excluded. The strong evidence supporting the use of adjuvant chemotherapy after radical surgery for NSCLC includes the finding that postoperative cisplatin-based chemotherapy significantly improves survival in stage IIB-III (8th edition of the TNM classification for lung and pleural tumors) NSCLC patients with hilar or mediastinal lymph node metastasis [6]. However, some cases with lymph node metastasis can avoid disease recurrence without adjuvant chemotherapy [9], while some cases without lymph node metastasis treated with radical surgery alone experience disease recurrence [10, 11]. Thus, predicting patients who could benefit from adjuvant chemotherapy remains difficult with the conventional diagnostic evidence and other histopathological prognostic factors reported, such as lymphatic vessel and blood vessel invasions [12].

The difficulty of predicting recurrence based on histopathological prognostic factors may be partly attributable to subjective judgment with vague qualitative expressions and the lack of a standard pathological assessment method [12]. Therefore, the limitations of conventional histopathological prognostic factors are thought to hinder retrospective validation by observers [13]. Accordingly, identification of novel, highly objective recurrence predictors is highly sought.

Lipid metabolism alterations in cancer cells, such as stimulation of lipid synthesis and lipid mobilization from adipose tissue, have been shown to influence several aspects of cancer phenotypes. For examples, cancer cell proliferation and invasion are promoted by enhanced synthesis of membrane lipids and cellular signalling lipids. Survival under oxidative stress and energy stress are promoted by membrane saturation and lipid droplet formation, respectively [14, 15]. Furthermore, some lipids have been suggested as prognostic factors in several cancer types [16–18]. In our previous study, increased sphingomyelin (SM)(d35:1) in lung adenocarcinoma (ADC) demonstrated excellent recurrence prediction ability (superior to histopathological factors) and was considered as a promising candidate predictor for recurrence after radical surgery [18]. The prediction ability of SM(d35:1) was considered excellent due to its high objectivity and is expected to overcome the limitation of histopathological prognostic factors in recurrence prediction. Following this result, identification of lipid candidate predictors for recurrence in lung squamous cell carcinomas (SQCCs), which is the major histological subtype of NSCLC behind only ADC, was anticipated [18].

Lung SQCC accounts for approximately 30% of NSCLC and is associated with poor clinical prognosis [19]. Recurrent lung SQCC cases after radical surgery can be treated with immune-checkpoint inhibitors regardless of their PD-L1 expression level [20, 21]. However, treatment modalities for SQCC are scarce compared to lung ADC, because lung SQCC lacks driver gene mutation targeted agents [22–25]. Accordingly, efficient application of adjuvant chemotherapy capable of improving prognosis is particularly crucial for lung SQCCs. Identification of novel recurrence predictors for lung SQCCs could enable accurate patient selection for adjuvant chemotherapy and lead to improved prognosis after radical surgery.

In this study, we explored candidate lipid predictors for recurrence by comparing lipidomes of recurrent and non-recurrent primary lung SQCC cases using liquid chromatography-tandem mass spectrometry (LC-MS/MS). Furthermore, we conducted a meta-analysis combining SQCC and ADC cohorts to compare lipidomic characteristics associated with recurrence between ADCs and SQCCs.

## Methods

### Patients and tissue samples

Retrospective frozen tissue samples of primary lung SQCC obtained from patients who received radical surgery with complete resection from January 2013 to December 2016 at Hamamatsu University Hospital were studied. Radical surgery was defined as follows: complete resection achieved by lobectomy with systematic lymph node dissection at stage I or II, and complete resection achieved by segmentectomy with or without lymph node sampling at stage I. Tissue samples of primary tumors were rapidly frozen in liquid nitrogen immediately after intraoperative collection and stored at − 80 °C until use. Histopathological diagnoses and pathological staging of the recruited cases were performed by experienced pathologists based on the World Health Organization criteria and the 8th edition of the TNM classification for lung and pleural tumors [26], respectively. Patient follow-up was performed with computed tomography (CT) of the body trunk and examination of blood squamous cell carcinoma antigen (SCC) and cytokeratin 19 fragments (CYFRA) [27, 28] every three months for the first two years, then, every six months until follow-up termination. The follow-up was continued until death or more than five years after surgery. If elevated SCC (≥ 2.5 ng/mL) or CYFRA (≥ 3.5 ng/mL) was observed without CT findings of recurrence, head magnetic resonance imaging and systemic positron emission tomography were performed to detect brain or bone metastasis.

We retrospectively reviewed clinical and histopathological records of the recruited tissue samples to determine eligibility. Cases with pathological stage I or II indicated for radical surgery were judged as eligible. Patients who received induction chemotherapy or radiotherapy were excluded.

We then assigned cases without and with recurrence to the non-recurrent and recurrent groups, respectively. Recurrence was defined as radiological imaging-based findings of distant or locoregional recurrence, whereas no recurrence was defined as the absence of distant or locoregional recurrence within the follow-up period. In the recurrent group, we excluded cases with recurrence of pleural dissemination, assuming the possible attribution to insufficient surgical margin. Ultimately, six and five cases were enrolled in the non-recurrent and recurrent groups, respectively, for the study.

### Histopathological evaluation

Three μm thick sections of paraffin-embedded tissue blocks were used for histopathological evaluation. SQCC was diagnosed according to the World Health Organization criteria. Hematoxylin-eosin (H&E) stained sections were evaluated for histological type, tumor size and lymph node metastasis. If necessary, immunohistochemistry was used to differentiate SQCC from ADC: squamous phenotype was confirmed by positive staining with one of the squamous markers (p40, p63, CK5/6) and negativity for TTF-1. D2–40 stain and Elastica van Gieson stain were used to evaluate lymphatic vessel invasion and blood vessel invasion, respectively.

### Chemicals for lipid extraction and LC-MS/MS analysis

Methanol, chloroform, glacial acetate, and ultrapure water used in lipid extraction were purchased from Wako Pure Chemical Industries (Osaka, Japan). In LC-MS/MS analysis, standard lipid levels were calibrated using 1,2-dilauroyl-sn-glycero-3-phosphatidylcholine (PC) (Avanti Polar Lipids, Alabaster, AL), PC (12:0_12:0), as an internal standard.

### Lipid extraction from the cancer tissue

Lipid extraction was performed as reported previously [18]. Briefly, we measured each tissue weight using a Sartorius analytical lab balance CPA224S (Sartorius AG, Göttingen, Germany) (Additional file 1, Supplemental Table 1). Subsequently, a modified Bligh-Dyer method [29] was performed for lipid extraction. The organic layers containing the extracted lipids were dried completely using miVac Duo LV (Genevac, Ipswich, England). After dissolving the dried lipids with 20 μL of methanol, we diluted 2 μL of the dissolved lipids with methanol proportional to the weight of the original tissue samples so that the PC (12:0_12:0) concentration was equalized among the samples.

### Lipid analysis by LC-MS/MS

LC-MS/MS analysis was performed as described previously [18]. Briefly, about 2 μL of the diluted lipid samples were separated on an Acclaim 120 C18 column (150 mm × 2.1 mm, 3 μm) (Thermo Scientific) and analyzed using a Q Exactive™ Hybrid Quadrupole-Orbitrap™ Mass Spectrometer (Thermo Scientific). We used the top 5 data-dependent MS2 methods with a resolution of 17,500 for identification based on spectral data recorded by an Xcalibur v3.0 Software (Thermo Scientific).

### Lipid identification and quantification

The spectral data acquired by the Xcalibur v3.0 Software was subjected to LipidSearch™ software version 4.2.13 (Mitsui Knowledge Industry, Tokyo, Japan) for identification and quantification of lipid ions. Identification was performed with parameter settings described previously [18]. For comparison analysis between the recurrent and non-recurrent groups, the identified lipid ions of the 11 cases were aligned with an RT tolerance of 0.5 min. Molecules that are annotated as redundant lipid ion names with different RT were regarded as independent structural isomers (annotated as “Duplication” in Additional file 2, Sheet 1).

### Data processing

Lipid intensities recorded in the Xcalibur v3.0 software and area values of lipid species identified by LipidSearch™ software were divided by the area values of the internal standard PC (12:0_12:0) for normalization.

For screening of candidate lipids for recurrence predictors, we compared lipidomes between the recurrent and non-recurrent groups by producing a volcano plot with -log_10_(*P*-value) for the vertical axis and log_2_(fold change) for the horizontal axis. Normalized area values of the lipid ions identified by LipidSearch™ software were used to construct the volcano plots. *P*-values of lipid ions were calculated using the Welch’s t-test by comparing area values between the recurrent and non-recurrent groups. Additionally, the fold change values for the lipid ions were evaluated by dividing the average area value of the recurrent group with that of the non-recurrent group. On the volcano plots, lipid ions with *P*-values of < 0.05 and fold change values of ≥2.0 or ≤ 0.5 were determined as significantly increased or decreased in the recurrent group.

To compare candidate lipid predictor levels between the non-recurrent and recurrent groups, intensity ratios of candidates were calculated as followings: normalized area values of a candidate lipid predictor in both groups were divided by the mean normalized area value of the candidate lipid predictor in the non-recurrent group.

Overall survival analyses for mRNA expressions of sphingomyelin synthase (SMS) and sphingomyelinase (SMase) were performed using lung SQCC-datasets in The Cancer Genome Atlas research network (http://cancergenome.nih.gov). Kaplan-Meier plots, hazard ratio, 95% confidence intervals and log-rank *P*-values were generated on Kaplan-Meier Plotter (https://kmplot.com/analysis/). Best cutoff values for discriminating good or poor prognosis groups were auto-selected.

Meta-analyses combining data from the lung SQCC cohort in this study and the lung ADC cohort in our previous report [18] were performed. In the lung ADC cohort, ADC phenotype was diagnosed with H&E stain and, if necessary, with positivity for TTF-1. LipidSearch™ software data sets of the SQCC and ADC cohorts were aligned with an RT tolerance of 0.8, then normalized by dividing with PC (12:0_12:0) area values. In the meta-analyses, total lipid levels and total SM levels were validated among the non-recurrent and recurrent groups of the two cohorts. Normalized lipid intensities recorded in the Xcalibur v3.0 software were used to compare total lipid levels. The total lipid level was calculated as the accumulation of normalized intensities of lipids in each case. Then, the intensity ratios of total lipid and total SM were calculated in the same manner used for the intensity ratios of the candidates. To visualize clustering of the intensity ratios of total lipid and total SM in the SQCC and ADC cohorts, heat maps of intensity ratios for lipid head groups and SM species were constructed. The lipid head group levels were calculated by accumulating normalized area values of the identified lipid ions with the same head species. After substituting area values of “0” with the trivial amount “0.0001” (to divide real numbers), intensity ratios of the lipid head groups and SM species were calculated as follows: area values were divided by the median lipid head group level or median SM species level of the non-recurrent group. Finally, we took log_2_ of the intensity ratios to display them in a heat-map using shinyheatmap (http://shinyheatmap.com/) [30]. The intensity ratio list of the lipid head groups and SM species are provided as Sheets 2 and 3 in Additional file 2, respectively.

### Statistical analysis

Associations between disease recurrence and patient clinical characteristics were evaluated using the Fisher exact test (categorical variables) or the Mann–Whitney U-test (for continuous variables). Recurrent-free survival (RFS) was determined as the time from radical surgery to the first disease recurrence or death; whichever comes first. The RFS curve of the recurrent group was obtained using the Kaplan–Meier method. The Welch’s t-test was used for creating the volcano plots and for comparing the candidate lipid predictor levels, total lipid intensity ratios and total SM intensity ratios between the non-recurrent and recurrent groups. The optimal cut-off ratios of candidate lipid predictors to discriminate the two groups were determined using receiver operating characteristic (ROC) curve analysis. The area under the ROC curves (AUCs) was calculated to evaluate the discrimination abilities of candidate lipid predictors. Spearman’s rank correlation analysis was used to evaluate the correlation between relative PC (12:0_12:0) levels and tissue weights or among final candidate lipid predictors. All statistical analyses, except for the t-tests, were carried out using R (The R Foundation for Statistical Computing, Vienna, Austria, version 3.6.2). The Welch’s t-test was performed using the “TTEST” function in Excel™ (Microsoft, Redmond, USA). For all the statistical analyses, *P*-values of < 0.05 were considered significant.

## Results

### Clinicopathological characteristics of the patient cohorts

Table 1 shows the clinicopathological characteristics of the patients enrolled in this study. Frozen primary tumor tissue samples from six non-recurrent and five recurrent cases were enrolled. Among the analyzed clinicopathological characteristics, differences in pleural invasion (Pl) (*P* = 0.002) and lymphatic vessel invasion (Ly) (*P* = 0.015) were statistically significant. The 1- and 2-year RFS rates of the recurrent group were 80 and 20% with median RFS time of 16 (range, 2–44) months, respectively (Additional file 1, Supplemental Fig. 1). The median follow-up time for the non-recurrent and recurrent groups was 56 (range, 47–75) and 23 (range, 4–54) months, respectively. Among the non-recurrent group patients, follow-up was terminated within 5 years in three patients (median, 48 months; range, 47–49 months); one of them was due to discontinuing follow-up, while the other patients were due to death from other diseases.

**Table 1:** Clinicopathological characteristics of the non-recurrent and recurrent groups

Characteristics	Non-recurrent (*n* = 6)	Recurrent (*n* = 5)	*P*-value
Median age (range)	70.5 (63–76)	78.0 (65–82)	0.261
Sex (male/female)	5/1	5/0	1.000
Smoking history (+/−)	6/0	5/0	1.000
Median Brinkman index (range)	890 (180–3000)	1770 (920–2000)	0.493
Pathological stage (I/II)	4/1	4/2	0.545
Median tumor size (mm) (range)	19 (12–34)	40 (10–43)	0.159
Degree of differentiation			0.437
Well	1	3	
Moderate	4	1	
Poor	1	1	
Lymph node metastasis (+/−)	1/5	1/4	1.000
Pleural invasion (+/−)	0/6	5/0	0.002*
Lymphatic vessel invasion (+/−)	1/5	5/0	0.015*
Blood vessel invasion (+/−)	3/3	5/0	0.182
Surgical procedure			0.455
Lobectomy	6	4	
Segmentectomy	0	1	
Adjuvant chemotherapy			
Indication (Stage IA3-IIB)	2	2	1.000
Received	0	1	1.000
Recurrent style			–
Locoregional	–	3	
Distant	–	2	

*Two histopathological prognostic factors were significant in the recurrent group

### Screening of candidate lipid predictors for recurrence

**Fig. 1:**
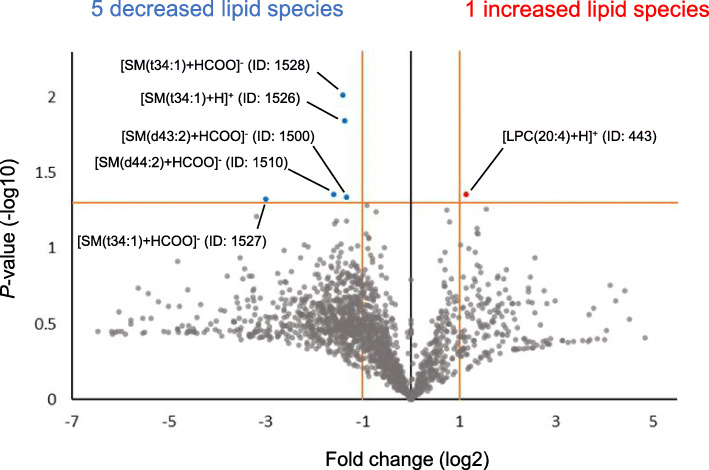
Volcano plots of 1745 identified lipid species for screening of candidate predictors. Each data point represents an individual lipid species. The relative amount of five lipid species was decreased (green symbols, FC ≤ 0.5 = left side of − 1 on the horizontal axis, *P*-value < 0.05 = 1.30 on the vertical axis), while one lipid species was increased (red symbol, FC ≥ 2.0 = right side of 1 on the horizontal axis, *P*-value < 0.05 = 1.30 on the vertical axis). These six lipid species with significant changes were selected as candidate predictors. Abbreviations: LPC, lysophosphatidylcholine; SM, sphingomyelin

A total of 1745 lipid species were identified from the 11 tissue samples by LC-MS/MS analysis. The full list of the 1745 identified lipid species is provided as Additional file 2 (Sheet 1). The quantified relative PC (12:0_12:0) levels, the internal standard, and tissue weights showed a strong positive correlation (Spearman’s rank correlation coefficient [rS] = 0.882, *P* < 0.001), which demonstrates high accuracy of the normalization procedure (Additional file 1, Supplemental Fig. 2). To screen candidate lipid predictors for recurrence, lipid ions with different levels between the two groups were identified by constructing a volcano plot (Fig. 1). The volcano plot identified six lipid ions with relative amounts significantly different between the two groups (fold change of ≥2.0 or ≤ 0.5; *P*-values, < 0.05). The number of lipid ions that decreased and increased in the recurrent group was five and one, respectively. Notably, all the decreased lipid ions were SM species, whereas the one increased lipid ion was lysophosphatidylcholine (LPC). These six lipid ions are presented with the following identity numbers (ID) in Additional file 2, Sheet 1: [SM(t34:1) + HCOO]^−^, 1528; [SM(t34:1) + H]^+^, 1526; [SM(d43:2) + HCOO]^−^, 1500; [SM(d44:2) + HCOO]^−^, 1510; [SM(t34:1) + HCOO]^−^, 1527; and [LPC (20:4) + H]^+^, 443.

**Fig. 2:**
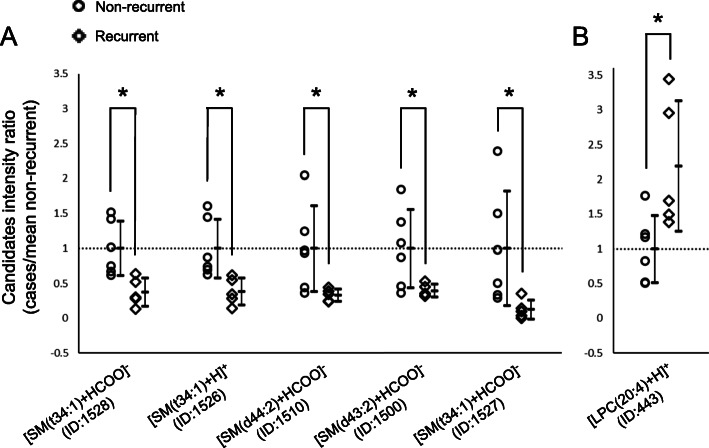
Comparison of intensity ratios of candidate lipids in the non-recurrent and recurrent groups (**A**: decreased and **B**: increased lipid species in the recurrent group). All the candidates demonstrated well-separated distributions between the two groups. Data are plotted as mean ± SD, along with individual data points. *P < 0.05, Welch’s t-test. Abbreviations: ID, identity number; LPC, lysophosphatidylcholine; SD, standard deviation; SM, sphingomyelin

We calculated and plotted intensity ratios (relative to the mean intensities of the non-recurrent group) for these six candidate lipid predictors (Fig. 2A and B) and compared their distribution between the two groups. All the candidate lipid predictors demonstrated well-separated distributions between the two groups.

Subsequently, we calculated the cut-off ratios and AUCs for these six lipid ions to determine their discrimination ability for the two groups. The top three lipid ions, according to the AUC values, were selected as final candidate predictors. Remarkably, all the final candidate predictors were SM species with the same fatty acid composition (t34:1) and demonstrated the following AUCs: [SM(t34:1) + H]^+^ (ID: 1526), 1.00; [SM(t34:1) + HCOO]^−^ (ID: 1528), 0.97; and [SM(t34:1) + HCOO]^−^ (ID: 1527), 0.93 (Table 2).

**Table 2:** AUC rank of candidate lipid predictors

Rank	Species	ID number	Cutoff ratio	AUC (95% CI)
1*	[SM(t34:1) + H]^+^	1526	0.623	1.00 (1.00–1.00)
2*	[SM(t34:1) + HCOO]^−^	1528	0.639	0.97 (0.87–1.00)
3*	[SM(t34:1) + HCOO]^−^	1527	0.140	0.93 (0.78–1.00)
4	[SM(d44:2) + HCOO]^−^	1510	0.440	0.90 (0.71–1.00)
4	[SM(d43:2) + HCOO]^−^	1500	0.530	0.90 (0.71–1.00)
4	[LPC(20:4) + H]^+^	443	1.390	0.90 (0.69–1.00)

*Lipids with the top three AUC were selected as final candidate predictors

Abbreviations: *AUC* area under the ROC curve, *CI* confidential interval, *ID* identity number, *LPC* lysophosphatidylcholine, *SM*,sphingomyelin

The MS/MS analyses (Additional file 1, Supplemental Fig. 3) indicated that a phosphocholine-head was detected for all the final candidate predictors. For [SM(t34:1) + HCOO]^−^ (ID: 1528) and [SM(t34:1) + HCOO]^−^ (ID: 1527), the phosphocholine-head was accompanied by the loss of a methyl group, and therefore SM(t34:1) with the loss of a methyl group was detected. In addition, fragmentation of the sphingoid base was detected for [SM(t34:1) + HCOO]^−^ (ID: 1528). Consequently, the annotations of the final candidate predictors by LipidSearch™ software were compatible with the results of MS/MS analyses.

### Comparison of recurrence prediction ability among the final candidate lipid predictors and histopathological prognostic factors

To compare the recurrence prediction ability between the screened candidate lipid predictors and histopathological prognostic factors (Pl and Ly, which were identified as significant factors for recurrence in Table 1), predictive parameters (sensitivity, specificity, and accuracy) were calculated for these factors (Table 3). For the lipid candidate predictors, the calculation was performed based on the cut-off ratios shown in Table 2.

**Table 3:** Comparison of sensitivity, specificity, and accuracy among the three final candidate predictors and histopathological prognostic factors

Predictors for recurrence	Sensitivity	Specificity	Accuracy
Candidate lipid predictors			
[SM(t34:1) + H]^+^ (ID: 1526)	1.00	1.00	1.00
[SM(t34:1) + HCOO]^−^ (ID: 1528)	1.00	0.83	0.91
[SM(t34:1) + HCOO]^−^ (ID: 1527)	0.80	1.00	0.91
Histopathological prognostic factors			
Pleural invasion	1.00	1.00	1.00
Lymphatic vessel invasion	1.00	0.83	0.91

Abbreviations: *ID* identity number, *SM* sphingomyelin

Among the predictors, [SM(t34:1) + H]^+^ (ID: 1526) and Pl showed the highest predictive value (1.00) among all the predictive parameters. [SM(t34:1) + HCOO]^−^ (ID: 1528) and Ly exhibited the same predictive values for all the parameters (sensitivity: 1.00, specificity: 0.83, accuracy: 0.91), while [SM(t34:1) + HCOO]^−^ (ID: 1527) demonstrated comparable predictive values (sensitivity: 0.80, specificity: 1.00, accuracy: 0.91). Based on these results, we surmise that all the candidate lipid predictors demonstrated excellent prediction performance that is equivalent to that of histopathological prognostic factors.

**Fig. 3:**
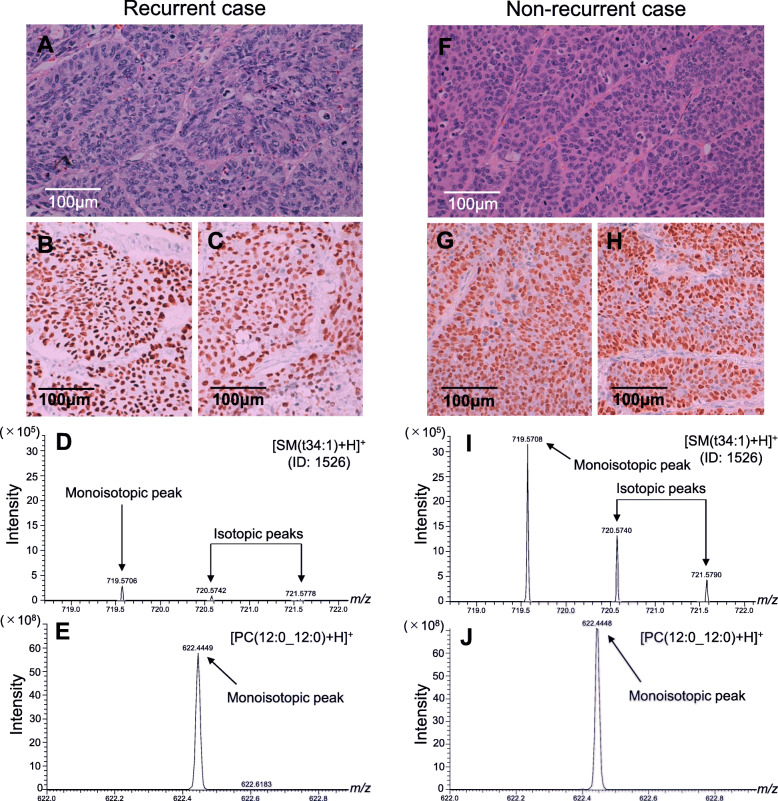
H&E images (magnification 200×) (**A**, **F**), IHC images (magnification 200×) of p40 (**B**, **G**) and p63 (**C**, **H**), and mass spectra of [SM(t34:1) + H]^+^ (ID: 1526) (**D**, **I**) and [PC (12:0_12:0) + H]^+^ (**E**, **J**) were compared between representative recurrent and non-recurrent cases. Lipid profiles of the two cases are from recurrent case 4 and non-recurrent case 6 (Additional file 2, sheet 1). The H&E and IHC images for both the recurrent and non-recurrent cases show moderately differentiated SQCC with diffuse positive staining of p40 and p63 demonstrating no obviously apparent differences between the two cases, whereas mass spectrum intensities of [SM(t34:1) + H]^+^ (ID: 1526) are markedly reduced in the recurrent case. The high mass resolution obtained in this study can be viewed as a monoisotopic peak and two isotopic peaks of [SM(t34:1) + H]^+^ (ID: 1526). Monoisotopic peaks for [SM(t34:1) + H]^+^ (ID: 1526) were normalized to the monoisotopic peaks for [PC (12:0_12:0) + H]^+^. Abbreviations: H&E, Hematoxylin-eosin; ID, identity number; IHC, immunohistochemistry; PC, phosphatidylcholine; SM, sphingomyelin; SQCC, squamous cell carcinoma

The histopathological images and mass spectrum of [SM(t34:1) + H]^+^ (ID: 1526), which showed the best prediction ability among the final candidate lipid predictors, were compared on representative recurrent and non-recurrent cases (Fig. 3). The H&E images in both recurrent and non-recurrent cases exhibited typical moderately differentiated SQCC with diffuse positive staining of p40 and p63 demonstrating no obviously apparently differences between the two cases. In contrast, the mass spectrum intensity of [SM(t34:1) + H]^+^ (ID: 1526) was markedly reduced in the recurrent case; therefore, [SM(t34:1) + H]^+^ (ID: 1526) appears to enable the highly objective prediction of recurrence.

SM is synthesized from ceramide by sphingomyelin synthase (SMS) that transfer the phosphocholine head group from phosphatidylcholine to ceramide and SM reconversion to ceramide is catalyzed by sphingomyelinase (SMase) [31]. Hence, we evaluated whether downregulated SMS or upregulated SMase expressions can be poor prognostic factors on lung SQCCs. However, overall survival analyses for mRNA expression levels of SMS and SMase showed no prognostic significance on lung SQCC datasets in The Cancer Genome Atlas research network (Additional file 1, Supplemental Fig. 4).

### A meta-analysis comparing lipidomic characteristics associated with recurrence between SQCC and ADC cohorts

To compare lipidomic characteristics associated with recurrence between SQCCs and ADCs, we conducted a meta-analysis combining the SQCC cohort of this study and the ADC cohort (non-recurrent group; *n* = 10, recurrent group; n = 10) from our previous study [18].

**Fig. 4:**
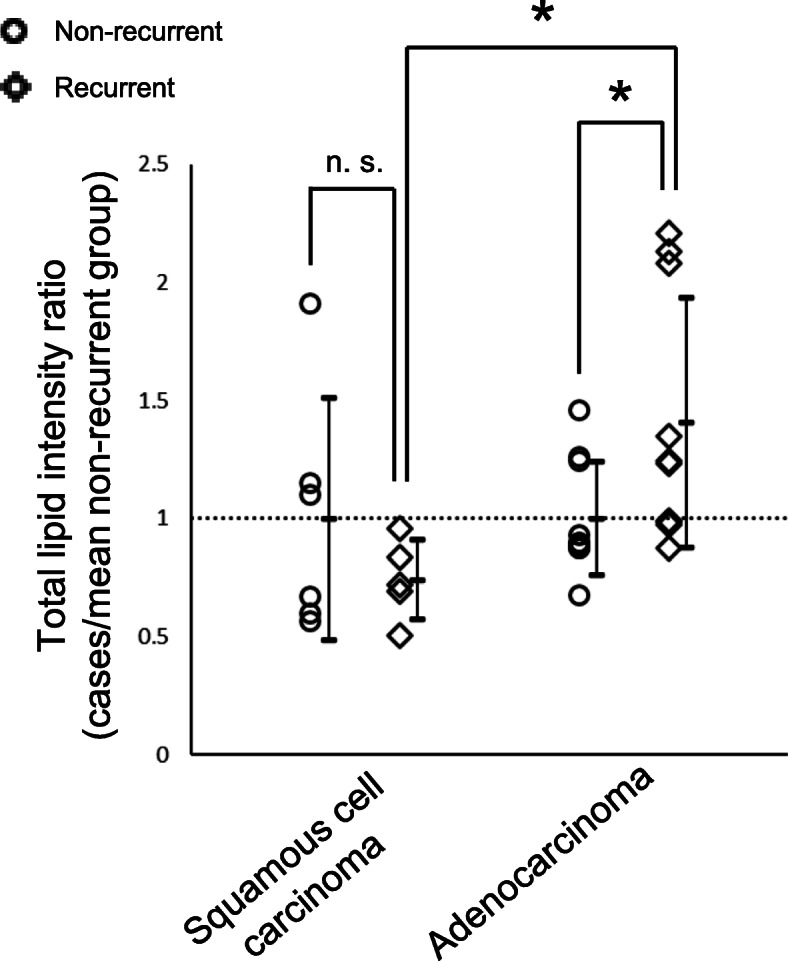
Differential meta-analysis of total lipid expression in the non-recurrent and recurrent groups of the SQCC and AD cohorts. Total lipid intensity ratios in the recurrent groups showed a tendency for a slight decrease in the SQCC cohort (mean intensity ratio = 0.74) and a significant increase in the ADC cohort (mean intensity ratio = 1.41), demonstrating a significant inverse trend between the two histological types. Data are plotted as mean ± SD, along with individual data points. *P < 0.05, Welch’s t-test. Abbreviations: ADC, adenocarcinoma; n.s., not significant; SM, sphingomyelin; SQCC, squamous cell carcinoma

We compared the intensity ratios of total lipid (cases/mean non-recurrent group) among recurrent and non-recurrent groups for both the ADC and SQCC cohorts (Fig. 4). In the SQCC cohort, the mean intensity ratio of total lipid in the recurrent group was 0.74 (weak decreasing trend; *P* = 0.313) while that of the ADC cohort was 1.41 (significant increase; *P* = 0.045). The opposite trend between the SQCC and ADC recurrent groups was significant (*P* = 0.018).

**Fig. 5:**
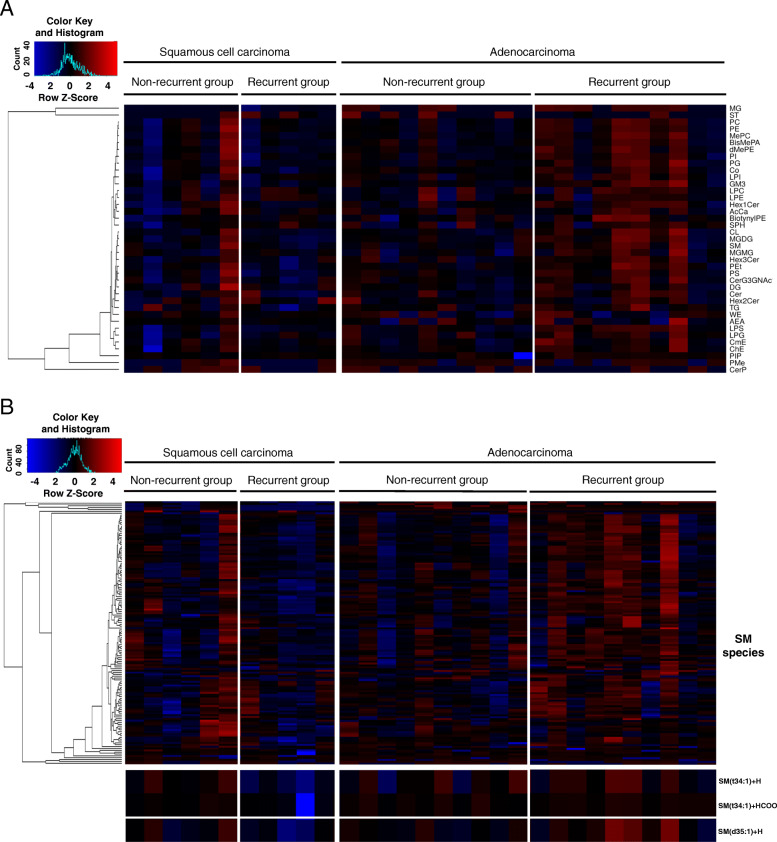
Lipid intensity ratio heat-maps showing the clustering of the intensity ratios of lipid head groups (**A**) and SM species (**B**) in the SQCC and ADC cohorts. The intensity ratios of lipid head groups show no prominent changes between the non-recurrent and recurrent groups of the SQCC cohort, whereas a broad range of lipid head groups is increased in the recurrent ADC group (A). The intensity ratios of SM species in the recurrent SQCC group demonstrated that lipid decreases were limited to SM(t34:1) and SM(d35:1). In contrast, the recurrent ADC group showed increases in a broad range of SM species. The full spellings of abbreviations for lipids are provided in Additional file 1, Sheets 2 and 3. Abbreviations: ADC, adenocarcinoma; SM, sphingomyelin; SQCC, squamous cell carcinoma

We then created a heat-map of intensity ratios for lipid head groups to visualize the opposite trend observed in the total lipid intensity ratios between recurrent SQCC and ADC. Figure 5A shows the total lipid intensity ratios clustered according to the head groups. In the majority of SQCC cases, no apparent difference was observed among the lipid head groups between the recurrent and non-recurrent groups, except for one non-recurrent case that exhibited increases in almost all of the lipid head groups. In contrast, widespread increases in lipid head groups were observed in the majority of the recurrent ADC cases.

Since SM species have been identified as candidate prognostic factors for both the ADC and SQCC cohorts, we compared the intensity ratios of total SM (cases/mean non-recurrent group) (Additional file 1, Supplemental Fig. 5) as performed for the total lipid intensity ratios. In the SQCC cohort, the mean intensity ratio of total SM in the recurrent group was 0.62 (weak decreasing trend; *P* = 0.320), while that of the ADC cohort was 1.67 (increasing trend; *P* = 0.052), showing a significant inverse trend (*P* = 0.005) between the SQCC and ADC recurrent groups. On a heat-map showing the intensity ratios of SM species (Fig. 5B), no apparent general difference in SM species between recurrent and non-recurrent groups was observed in the SQCC cohort. In contrast, the intensity ratios of the candidate recurrence predictors for SQCC identified in this study ([SM(t34:1) + H]^+^, [SM(t34:1) + HCOO]^−^) demonstrated consistent decreases in the SQCC recurrent group relative to the non-recurrent group, while the candidate recurrence predictor for ADC identified in our previous study ([SM(d35:1) + H]^+^) showed a tendency similar to the predictors for SQCC. The one exceptional non-recurrent SQCC case showed broad increases in SM species that were consistent with the observed increases in lipid head groups. In brief, SM species showing consistent decreases in the SQCC recurrent group was narrowly limited. In contrast, broad increases of SM species were demonstrated in five of the ten total cases in the recurrent ADC group. The candidate recurrence predictor for ADC also showed relatively consistent increases in the recurrent group, while the candidate recurrence predictors for SQCC demonstrated a tendency similar to the predictor for ADC.

In summary, the lipidomic characteristics associated with recurrence were as follows: decreases in a narrowly limited group of SM species [SM(t34:1) and SM(d35:1)] in the SQCC cohort, which is in opposition to the broad increases in lipid species in the ADC cohort.

## Discussion

In this study, we screened candidate lipid predictors for lung SQCC recurrence after radical surgery and found that decreased SM(t34:1) was a promising candidate predictor showing excellent prediction performance that was equivalent to that of histopathological prognostic factors in this small cohort.

Importantly, our screening process resulted in the identification of five lipid species that were decreased in the recurrent group, which were all identified as SM species. Furthermore, all of the top three candidates were SM(t34:1). SM is a major bioactive component of lipid rafts in the cellular membrane and regulates cancer cell proliferation, migration, survival, and chemo-resistance [31, 32]. Several SM species are decreased in cancer tissue compared to normal lung tissue in NSCLC, including SQCC, due to high consumption of lipids in cancer tissue [33, 34]. Notably, SM (34:1) in cancer tissues is reportedly decreased relative to normal tissue in NSCLC and prostate cancer [33, 35]. In our study, because a significant decrease in SM(t34:1) was observed in the recurrent group, despite only a slight decrease in the total SM levels, it raised the possibility that SM(t34:1) may have biological roles in cancer progression. Among the final candidate predictors, [SM(t34:1) + H]^+^ (ID: 1526) and [SM(t34:1) + HCOO]^−^ (ID: 1528) exhibited an extremely high positive correlation (rS = 0.991, *P* < 0.001) (Additional file 1, Supplemental Fig. 6A) with an RT difference (0.006 min) that is small enough to ignore (Additional file 2, Sheet 1). Therefore, these two lipids were considered to be identical species with different ion adducts. In contrast, [SM(t34:1) + HCOO]^−^ (ID: 1527) was thought to be an isomer showing significant positive correlation and relatively large RT differences with [SM(t34:1) + H]^+^ (ID: 1526) (rS = 0.800, *P* = 0.005, RT difference = 2.967) and [SM(t34:1) + HCOO]^−^ (ID: 1528) (rS = 0.773, *P* = 0.008, RT difference = 2.961) (Additional file 1, Supplemental Fig. 6B and 6C, Additional file 2, Sheet 1).

The three final candidate lipid predictors demonstrated near-perfect prediction performance that was equivalent to that of the histopathological prognostic factors, Pl and Ly. Because SM species regulate cancer cell invasiveness [31], the similar prediction performance of our candidate lipid predictors and the two histological prognostic factors, which reflect the invasiveness of the cancer tissue, was considered to be plausible. However, the observed ideal prediction performance of the candidate lipid predictors may be an artifact of the small sample size. Similarly, the near-perfect prediction abilities demonstrated by the histopathological prognostic factors also suggest an influence of the small sample size, since they showed higher performance than in clinical practice [12, 36]. Therefore, the predictive performance of the candidate lipid predictors in a large cohort is assumed to be lower than in this study. Nonetheless, as the difference in SM(t34:1) levels was prominent between recurrent and non-recurrent cases with apparently no significant difference in the histopathological images (Fig. 3), SM(t34:1) is considered to have an advantage in assisting highly objective recurrence prediction.

In the overall survival analyses using the lung SQCC datasets in The Cancer Genome Atlas research network, the mRNA expression levels of the SMS and SMase were shown to have no prognostic influence. Our result that the total SM intensity showed an only weak decreasing trend in the recurrent group of our study cohort (Additional file 1, Supplemental Fig. 5) does not contradict if the SMS and SMase mRNA expression levels do not differ significantly between the recurrent and non-recurrent groups.

In the meta-analysis comparing the SQCC and AD cohorts, total lipid and SM levels of the recurrent groups showed a slight tendency to decrease in the SQCC cohort, contrary to the significant increases in the ADC cohort, demonstrating opposing trends between the two histological types. Generally, lipid synthesis and uptake are activated in cancer tissues to supplement lipid consumption caused by rapid cell proliferation [37], while some studies have shown decreases in lipid storage along with accelerated lipid consumption during cancer progression or high malignancy acquisition [17, 38]. The opposing trend in total lipid levels between the SQCC and ADC recurrent groups may be explainable by the following hypothesis: lipid replenishment is sufficient but lipid consumption is a rate-determining step for acquiring recurrence potential in SQCCs, while lipid consumption is sufficient but lipid replenishment is a rate-determining step for acquiring recurrence potential in ADCs. Concerning the role of SM in cancer progression, previous studies have reported conflicting results among different cancer types; the promotion of cancer progression has been shown in leukemia and cervical cancer [39, 40], while anti-tumor effects have been demonstrated in colon cancer and glioma [41, 42]. The reciprocal trend in total SM between the ADC and SQCC cohorts in our study may be explained by the proposal that SM has opposing biological roles in cancer progression according to the cancer type. Alternatively, the decreased total SM levels in the recurrent SQCC group may also be consistent with the evidence that upregulated SM consumption is triggered by inflammation. Lung SQCCs are strongly correlated with smoking, which evokes inflammation in lung tissue resulting in activation of the arachidonic acid pathway [43]. In our study cohort, smoking history and the Brinkman index were significantly frequent and high in the SQCC group compared to the ADC group (Additional file 1, Supplemental Table 2). As a result, eicosanoids, the metabolic product of arachidonic acid, might have activated SMase, which metabolizes and consumes SM [44].

Our study has several limitations. First, this retrospective study was performed on a small sample size because frozen tissue samples that meet our inclusion criteria were scarce; therefore, the identified lipid predictors cannot be considered more than “candidates” requiring further validation. Furthermore, because a large number of lipid candidates (1745 species) were validated as variables in a small number of research subjects (11 cases), candidates that show near-perfect prediction performance may tend to be found. Further studies on a large cohort should be performed to validate the candidate predictors identified in this study as robust predictors. Then, subsequent retrospective and prospective studies evaluating the correlation between the candidate lipid predictors and efficacy of adjuvant chemotherapy may enable utilizing the efficient patient selection for adjuvant chemotherapy. Second, the follow-up periods of three patients in the non-recurrent group were shorter than five years (median, 48 months; range, 47–49 months) due to discontinuing follow-up or death from other diseases; therefore, recurrence later than the follow-up termination might be concealed. However, considering that more than 80% of recurrences occur within the first two years [10], the possibility of recurrences later than our follow-up termination was assumed to be low. Third, because normal lung tissue samples adjacent to the cancer tissue samples were lacking, differences in SM levels between the normal lung tissue and the cancer tissue, or between the normal lung tissue of the recurrent group and that of the non-recurrent group was not compared. Fourth, LC-MS/MS is not a standard diagnostic modality for postoperative lung cancer patients in the present circumstances. Therefore, introducing LC-MS/MS screening may be costly on its initial investment. However, LC-MS/MS has been utilized for high-speed cancer screening using AminoIndex® as an optional modality in recent years [45, 46]. If LC-MS/MS screening enables efficient postoperative patient selection for adjuvant chemotherapy, medical expense reduction that surpasses the initial investment may be achieved by suppressing recurrence treatment and unnecessary application of adjuvant chemotherapy. Fifth, recurrence prediction by inspecting the surgical specimen is not able to monitor chronologically the recurrence after surgery. Future studies identifying lipid predictors in blood plasma samples that enable chronological monitoring of disease recurrence are expected.

## Conclusions

We found that decreased SM(t34:1) is a promising candidate predictor for lung SQCC recurrence after radical surgery. Decreases in a limited number of SM species, including SM(t34:1), in SQCCs and increases in a broad range of lipid species in ADCs were suggested as lipidomic characteristics associated with recurrence. A further large cohort study is mandatory to validate the candidate predictors identified in the present study as robust predictors.

## Supplementary Information

**Additional file 1: Table 1.** Weights of the frozen tissue samples. **Figure 1**. Recurrence-free survival curve of the recurrent group. **Figure 2**. Correlation between relative phosphatidylcholine (12:0_12:0) levels and sample tissue weights. **Figure 3**. Tandem mass spectrometry analyses of the final three candidate predictors: [sphingomyelin (SM)(t34:1) + H]^+^ (ID: 1526) (A), [SM(t34:1) + HCOO]^−^ (ID: 1528) (B), and [SM(t34:1) + HCOO]^−^ (ID: 1527) (C). **Figure 4**. Overall survival curves for mRNA expression levels of sphingomyelin synthase and sphingomyelinase on lung squamous cell carcinoma. **Figure 5**. Comparison of total sphingomyelin intensity ratios among the non-recurrent and recurrent groups of the squamous cell carcinoma and adenocarcinoma cohorts. **Fig. 6**. Correlations among the final three candidate predictors. **Table 2**. Comparison of smoking history and the Brinkman index between the squamous cell carcinoma and adenocarcinoma groups

**Additional file 2: ** Sheet 1. The full list of area values for the 1745 lipid species identified. Sheet 2. List of the intensity ratios according to the lipid head groups. Sheet 3. List of the intensity ratios according to the sphingomyelin species

## Data Availability

The dataset supporting the conclusions of this article is included within the Additional Files.

## Abbreviations

ADC: Adenocarcinoma
AUC: Area under the ROC curves
H&E: Hematoxylin-eosin
ICI: Immune-checkpoint inhibitor
ID: Identity numbers
LC-MS/MS: Liquid chromatography-tandem mass spectrometry
LPC: Lysophosphatidylcholine
Ly: Lymphatic vessel invasion
NSCLC: Non-small cell lung cancer
PC: Phosphatidylcholine
Pl: Pleural invasion
RFS: Recurrent-free survival
ROC: Receiver operating characteristic
rS: Spearman’s rank correlation coefficient
RT: Retention time
SM: Sphingomyelin
SQCC: Squamous cell carcinoma
