# Supplementary material for: Decreased sphingomyelin (t34:1) is a candidate predictor for lung squamous cell carcinoma recurrence after radical surgery: a case-control study
Source: BMC Cancer. 2021 Nov 17;21:1232. doi: 10.1186/s12885-021-08948-5 (PMC8597230; doi:10.1186/s12885-021-08948-5)
Supplement: Supplementary file 1 — Additional file 1: Table 1. Weights of the frozen tissue samples. Figure 1. Recurrence-free survival curve of the recurrent group. Figure 2. Correlation between relative phosphatidylcholine (12:0_12:0) levels and sample tissue weights. Figure 3. Tandem mass spectrometry analyses of the final three candidate predictors: [sphingomyelin (SM)(t34:1) + H]+ (ID: 1526) (A), [SM(t34:1) + HCOO]− (ID: 1528) (B), and [SM(t34:1) + HCOO]− (ID: 1527) (C). Figure 4. Overall survival curves for mRNA expression levels of sphingomyelin synthase and sphingomyelinase on lung squamous cell carcinoma. Figure 5. Comparison of total sphingomyelin intensity ratios among the non-recurrent and recurrent groups of the squamous cell carcinoma and adenocarcinoma cohorts. Fig. 6. Correlations among the final three candidate predictors. Table 2. Comparison of smoking history and the Brinkman index between the squamous cell carcinoma and adenocarcinoma groups [file 12885_2021_8948_MOESM1_ESM.pdf]

Group	Case	Tissue weight (mg)
Recurrent	1	20
	2	15.2
	3	69.6
	4	20.6
	5	19.8
Non-recurrent	1	13.5
	2	24.4
	3	20.7
	4	9
	5	16.7
	6	1.5

Supplemental Table 1. Weights of the frozen tissue samples.

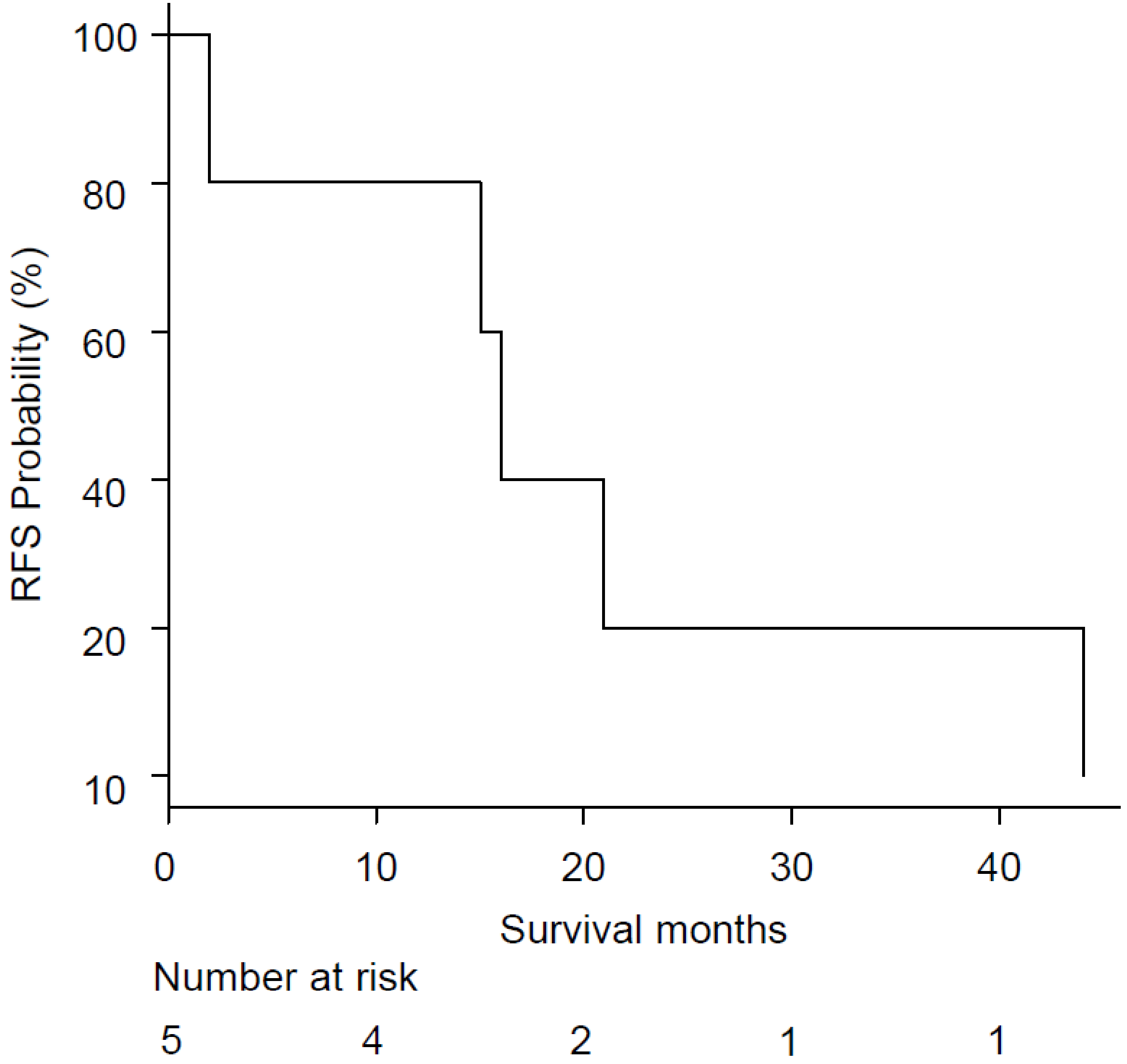

Supplemental Figure 1. Recurrence-free survival curve of the recurrent group.

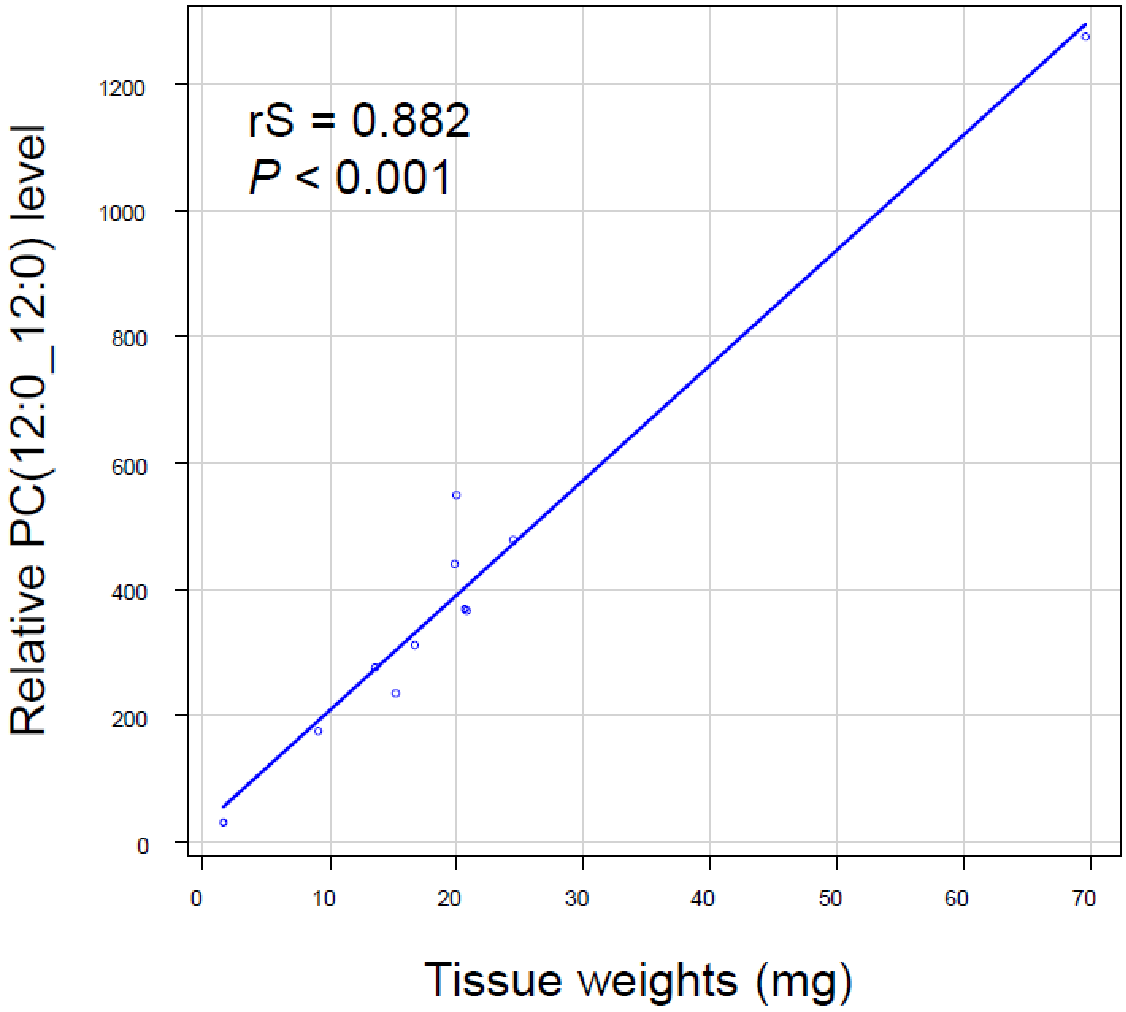

Supplemental Figure 2. Correlation between relative PC(12:0_12:0) level and sample tissue weights.

Abbreviations: PC, phosphatidylcholine; rS, Spearman's rank correlation coefficient.

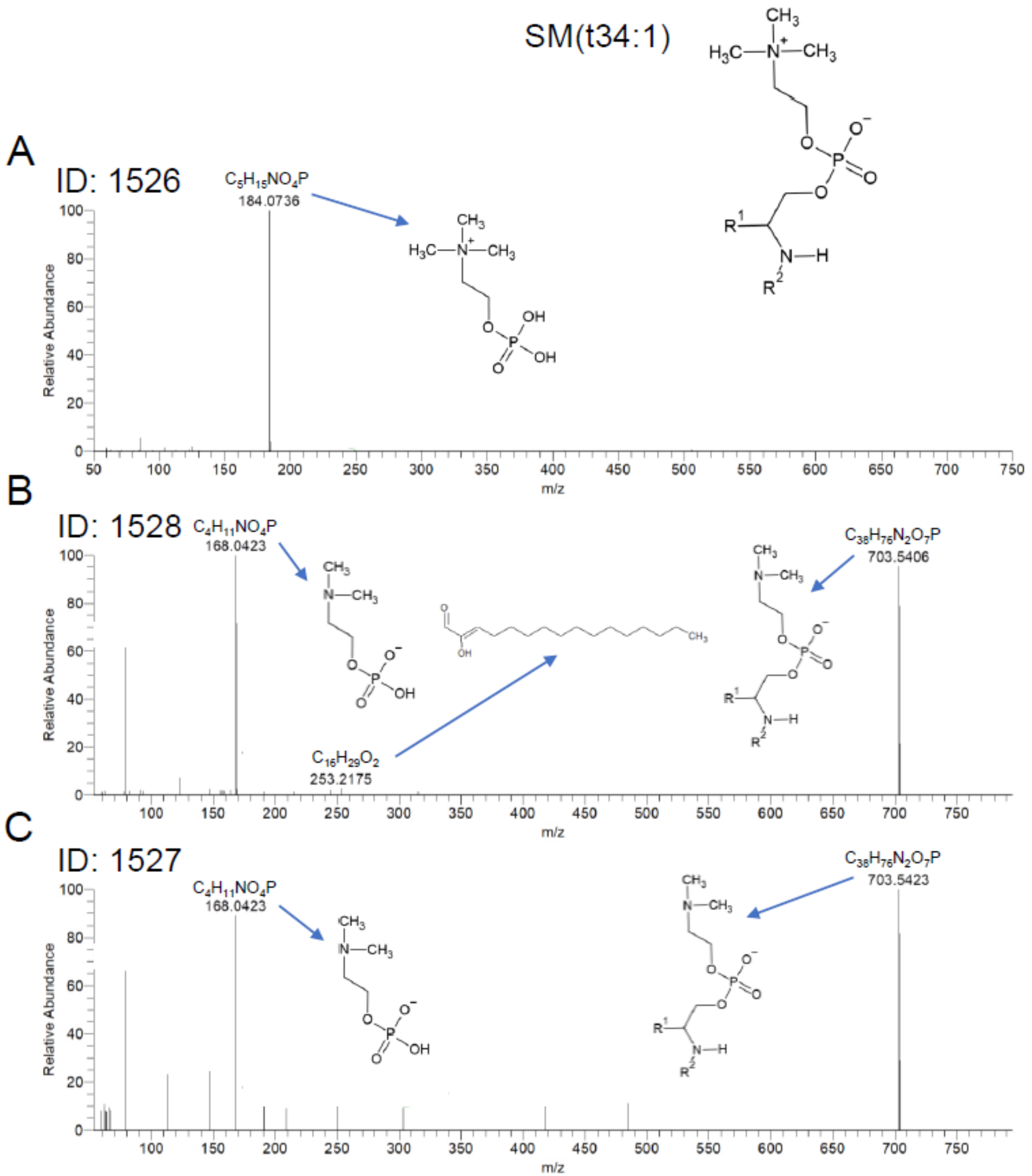

Supplemental Figure 3. Tandem mass spectrometry analyses of the final three candidate predictors: [sphingomyelin (SM)(t34:1)+H]+ (ID: 1526) (A), [SM(t34:1)+HCOO]- (ID: 1528) (B), and [SM(t34:1)+HCOO]- (ID: 1527) (C).
$C_5H_{15}NO_4P$, phosphocholine head; $C_4H_{11}NO_4P$, phosphocholine head with a loss of a methyl group; $C_{38}H_{75}N_2O_7P$, SM(t34:1) with a loss of a methyl group; $C_{15}H_2O_2$, sphingoid base.

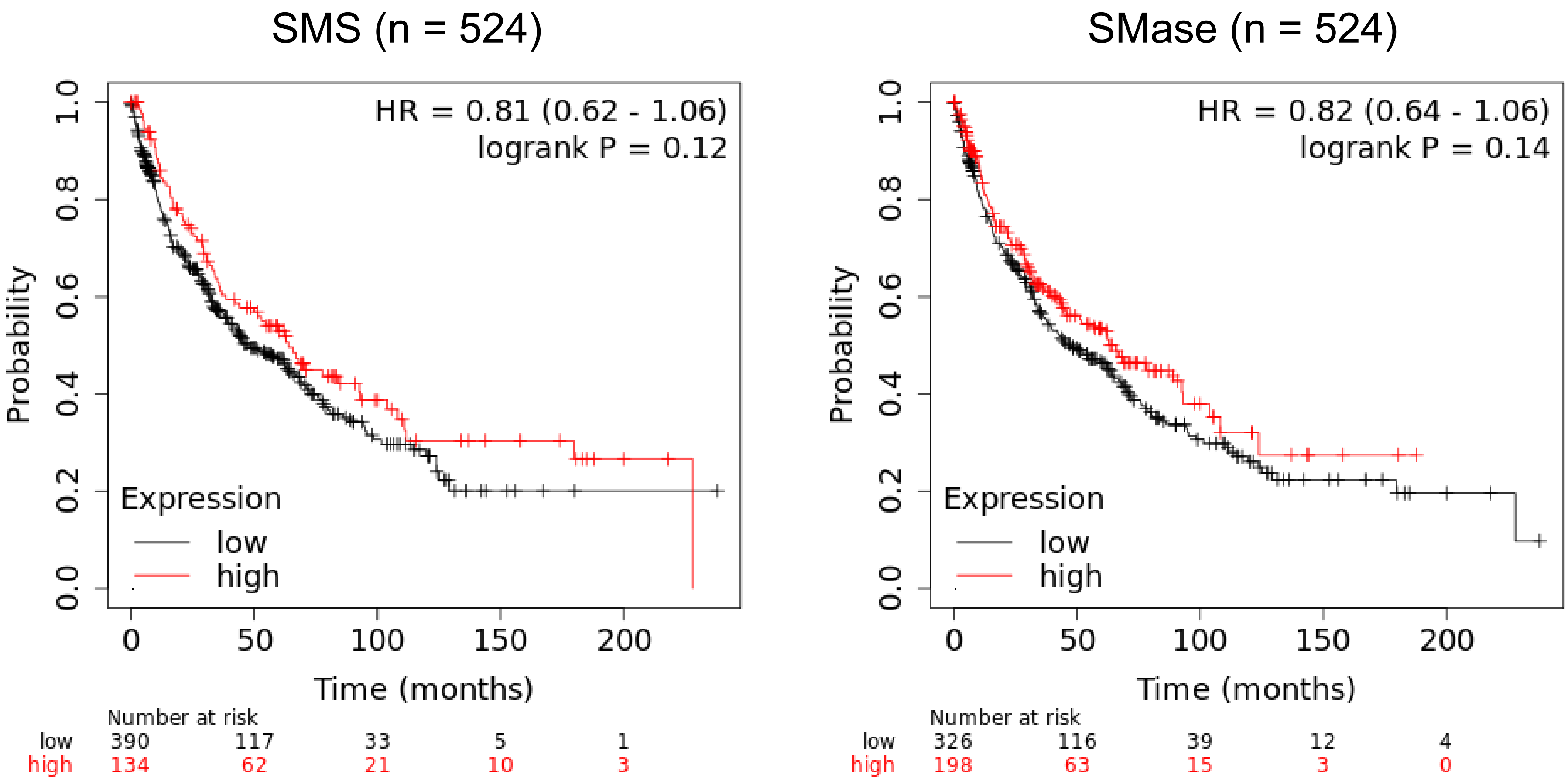

Supplemental Figure 4. Overall survival curves for mRNA expression levels of SMS and SMase on lung SQCC. High and low mRNA expression groups are shown as red and black lines, respectively. HR, 95% confidence intervals and log-rank *P*-values are presented. Abbreviations: HR, hazard ratio; SMase, sphingomyelinase; SMS, sphingomyelin synthase; SQCC, squamous cell carcinoma.

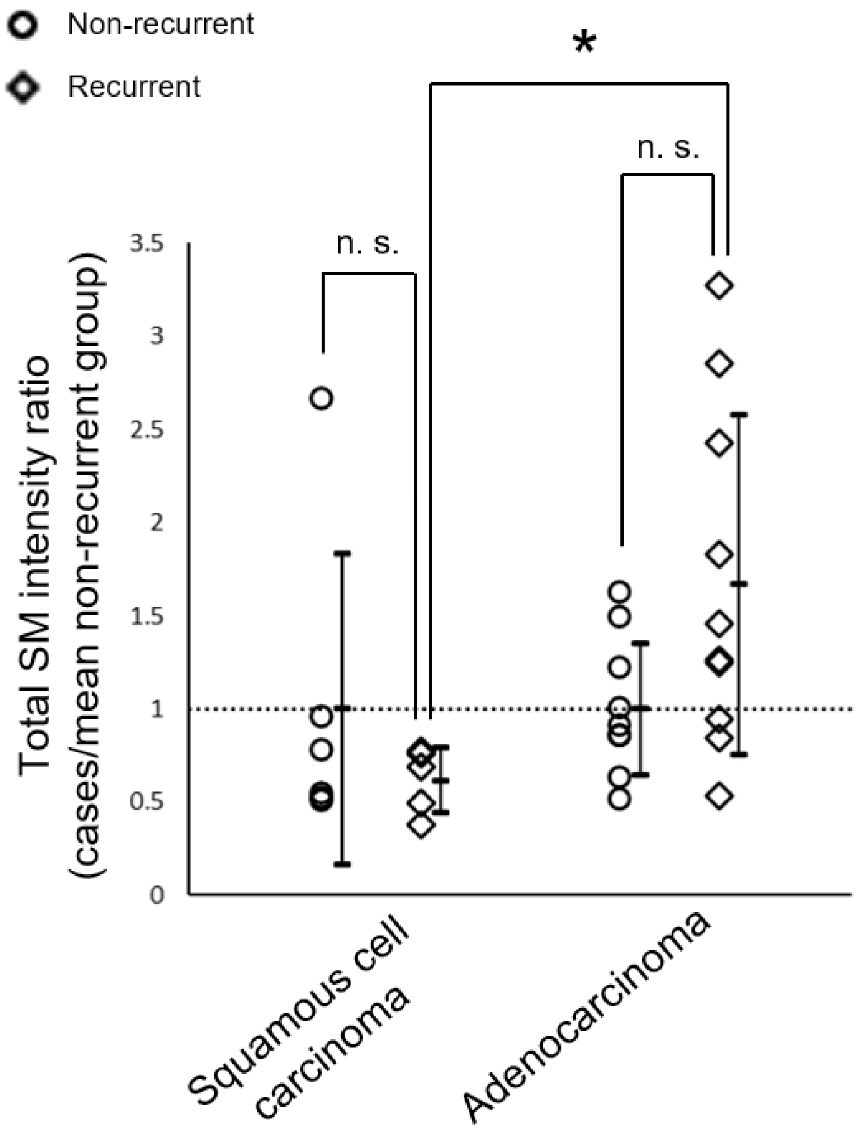

Supplemental Figure 5. Comparison of total SM intensity ratio among the non-recurrent and recurrent groups of the squamous cell carcinoma and adenocarcinoma cohorts. Asterisks indicate significant differences (*P < 0.05) in the Welch's t-test. Abbreviations: n.s., no significance; SM, sphingomyelin.

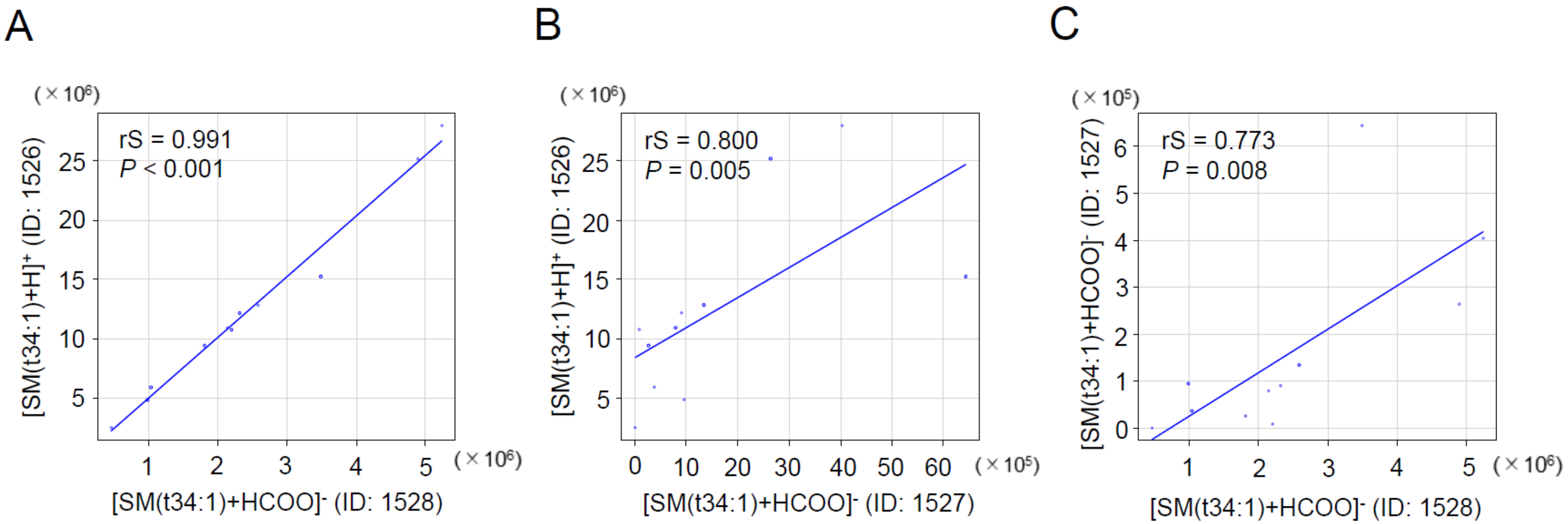

Supplemental Figure 6. Correlation among the final three candidate predictors. Abbreviations: rS, Spearman's rank correlation coefficient; SM, sphingomyelin.

	SQCC (n = 11)	ADC (n = 20)	*P*-value
Smoking history (+/-)	11/0	13/7	0.033*
Median Brinkman index (range)	1240 (180-3000)	538 (0-2100)	0.012*

Supplemental Table 2. Comparison of smoking history and the Brinkman index between the SQCC and ADC groups.
Asterisks indicate significant differences (*P < 0.05) in the Fisher exact test (Smoking history) and the Mann-Whitney U-test (Brinkman Index) between the two groups.
Abbreviations: ADC, adenocarcinoma; SQCC, squamous cell carcinoma.
